# ELAVL1 promotes prostate cancer progression by interacting with other m6A regulators

**DOI:** 10.3389/fonc.2022.939784

**Published:** 2022-08-01

**Authors:** Zhonglin Cai, Huan Xu, Gang Bai, Hanjing Hu, Di Wang, Hongjun Li, Zhong Wang

**Affiliations:** ^1^ Department of Urology, Shanghai Ninth People’s Hospital, Shanghai Jiaotong University School of Medicine, Shanghai, China; ^2^ Center for Reproductive Medicine, Cheeloo College of Medicine, Shandong University, Jinan, China; ^3^ Department of Biochemistry and Molecular Biology, School of Medicine, Nantong University, Nantong, China; ^4^ Department of Molecular Pathology, Fujian Medical University Cancer Hospital, Fujian Cancer Hospital, Fuzhou, China; ^5^ Department of Urology, Peking Union Medical College Hospital, Peking Union Medical College, Chinese Academy of Medical Sciences, Beijing, China

**Keywords:** prostate cancer, ELAVL1, m6A, m6A regulators, interaction

## Abstract

N6-Methyladenosine (m6A) imbalance is an important factor in the occurrence and development of prostate cancer (PCa). Many m6A regulators have been found to be significantly dysregulated in PCa. ELAVL1 is an m6A binding protein that can promote the occurrence and development of tumors in an m6A-dependent manner. In this study, we found that most m6A regulators were significantly dysregulated in PCa, and some m6A regulators were associated with the progression-free interval. Mutations and copy number variations of these m6A regulators can alter their expression. However, ELAVL1 mutations were not found in PCa. Nevertheless, ELAVL1 upregulation was closely related to PCa proliferation. High ELAVL1 expression was also related to RNA metabolism. Further experiments showed that ELAVL1 interacted with other m6A regulators and that several m6A regulatory mRNAs have m6A sites that can be recognized by ELAVL1. Additionally, protein–protein interactions occur between ELAVL1 and other m6A regulators. Finally, we found that the dysregulation of ELAVL1 expression occurred in almost all tumors, and interactions between ELAVL1 and other m6A regulators also existed in almost all tumors. In summary, ELAVL1 is an important molecule in the development of PCa, and its interactions with other m6A regulators may play important roles in PCa progression.

## Introduction

N6-Methyladenosine (m6A) is an RNA modification in which the 6th hydrogen atom of adenine is substituted by a methyl group (1). m6A is present in almost all types of RNA, including mRNA, tRNA, rRNA and noncoding RNA, and approximately half of mRNAs in eukaryotic cells have m6A sites (1, 2). mRNA is dynamically modified by methylation and demethylation at m6A sites, which ultimately impacts mRNA metabolism depending on the recognition of m6A binding proteins (3). Previous studies have reported that the dynamic regulation of m6A is involved in adipogenesis, brain neurodevelopment, hematopoietic stem cell differentiation, circadian rhythm maintenance, immune function regulation, and spermatogenesis (4–9). An imbalance in m6A can lead to the occurrence of various diseases, including tumors, obesity, and cardiovascular diseases (10–12).

Several studies have shown that m6A dysregulation is associated with tumorigenesis and cancer progression (13–16). The expression of multiple m6A regulators, including METTL3, FTO, ALKBH5, and YTHDF3, is significantly changed in different tumors, including lung cancer, osteosarcoma, gastric cancer, melanoma, and cholangiocarcinoma (17–21). These changes in m6A regulators affect the biological functions of tumor proliferation, migration, invasion, and cell cycle progression through m6A. Prostate cancer (PCa) is a common tumor in elderly men. Similar to the case of other tumors, in PCa, the level of m6A is significantly higher than that in normal prostate tissue, and the expression of some m6A regulators, including FTO, METTL3, IGF2BP2, and YTHDF2, is downregulated (22–26). The dysregulated m6A regulator-mediated abnormal RNA metabolism pathways, such as RNA stability, have been studied in detail.

ELAVL1 is an important RNA-binding protein, and numerous studies have found that it is highly expressed in different tumors, including lung cancer, liver cancer, and pancreatic cancer, and that it promotes tumor occurrence and development (27–29). ELAVL1 expression is also related to resistance to chemotherapy (30–32) and radiotherapy (33, 34). In recent years, ELAVL1 was found to be an m6A regulator that acts as a reader, binding to RNAs with m6A sites and increasing the stability of those RNAs (33, 35). In addition, ELAVL1 has been reported to synergistically promote RNA stabilization by binding to molecules such as YTHDC1 and IGF2BP1 in tumors (36, 37). Furthermore, previous studies have reported that ELAVL1 is highly expressed in PCa and promotes its development (38, 39). However, the interaction of ELAVL1 with other m6A regulators in PCa remains to be studied.

In the present study, we analyzed the multiomic signature of m6A regulators in PCa. ELAVL1 was found to be significantly correlated with certain m6A regulators. At the same time, ELAVL1 was related to the regulation of several m6A regulators through m6A, and it had obvious protein–protein interactions with other m6A regulators. Overall, our study revealed the interactions between ELAVL1 and other m6A regulators in PCa and broadened our knowledge of the m6A regulatory network in PCa.

## Materials and methods

### Cell lines and culture

The human PCa cell lines LNCaP and PC-3 and the human normal prostate epithelial cell line RWPE-1 were purchased from the Cell Resource Centre of Peking Union Medical College (Beijing, China). PC-3 cells were cultured in DMEM (Gibco; 10566016) containing 10% fetal bovine serum (FBS), LNCaP cells were cultured in 1640 medium (Gibco; C11875500BT), and RWPE-1 cells were cultured in Prostate Epithelial Cell Medium (ScienCell, 4411).

### Adenovirus infection

ELAVL1 knockdown (shELAVL1 sequence- sense: 5’-AATTCGTACCAGTTTCAATGGTCATAATTCAAGAGATTATGACCATTGAAACTGGTATTTTTTG-3’; antisense: 5’-GATCCAAAAAATACCAGTTTCAATGGTCATAATctcttgaaTTATGACCATTGAAACTGGTACg-3) and control adenoviruses were purchased from Hanbio Co., Ltd. (Shanghai, China). Adenoviral infection was performed according to the recommended protocol. Briefly, control and knockdown adenoviruses (MOI=100) were quantified and diluted in serum‐free DMEM. Subsequently, the adenovirus mixtures were added to the cultured plates containing DMEM. The supernatants were discarded after 8 h and replaced with standard DMEM containing 10% FBS for the next assay.

### RNA extraction and RT–qPCR

Total RNA was extracted with TRIzol™ Reagent according to the manufacturer’s instructions (Invitrogen, 15596026). Precipitated RNA was reverse transcribed into cDNA using ReverTra Ace qPCR RT Master Mix with gDNA Remover (TOYOBO, FSQ-301), and qPCR was performed using THUNDERBIRD SYBR qPCR Mix (TOYOBO, QPS-201). *ELAVL1* was amplified with the forward primer TAAGGTGTCGTATGCTCGCC and reverse primer CGGATAAACGCAACCCCTCT. *GAPDH*-forward: 5’- TCAAGGCTGAGAACGGGAAG-3’, *GAPDH*-reverse: 5’- TCGCCCCACTTGATTTTGGA-3’.

### Immunohistochemistry

PCa tissue chip sections (4 µm) obtained from Shanghai Outdo Biotech Company were used for IHC staining. Sections were dewaxed with xylene and rinsed sequentially with 100%, 95%, and 75% ethanol. Then, the sections were heated in citric acid at 95°C for 10 min for antigen retrieval. Subsequently, endogenous catalase was blocked by treatment with 3% hydrogen peroxide at room temperature for 10 min. Sections were then incubated with primary antibodies, followed by horseradish peroxidase-labeled secondary antibodies. Finally, the sections were stained with diaminobenzidine and counterstained with hematoxylin.

### Cell proliferation assay

Cell proliferation assays were performed using Cell Counting Kit-8 (Beyotime, C0037). Cells were seeded in 96-well plates with 3000 cells per well and incubated with 10% CCK-8 medium for 1 h at 0 h, 24 h, 36 h, 48 h, and 72 h after seeding. The absorbance at 450 nm was measured with a multimode reader (Varioskan Flash, Thermo).

### Coimmunoprecipitation and mass spectrometry

Coimmunoprecipitation was performed using an Immunoprecipitation Kit (Beyotime, P2179S) according to the manufacturer’s instructions. Briefly, the cells were lysed and then incubated with the primary antibody at 4° overnight; then, Protein A+G beads were added to the cell lysate and primary antibody mixture for 1 h at room temperature, and the beads were washed with 1× TBS. After that, 1×SDS–PAGE was performed to elute protein from the beads. As previously described (40), the eluate was digested into peptides, alkylated and desalted. It was then injected into an Ultimate 3000 RSLCnano system (Thermo Fisher Scientific) for mass spectrometry analysis. MaxQuant version 1.5.2.8 software was used for protein identification and quantification by intensity-based absolute quantification (iBAQ) values (41). CRAPome analysis was used to identify and remove nonspecifically bound proteins, as previously described (42).

### RNA immunoprecipitation assay and sequencing

Cells were collected and lysed with nondenatured lysate on ice for 30 min. After centrifugation, the supernatant was collected and quantified with BCA protein (Thermo Scientific, 23227). An appropriate amount of supernatant was mixed with primary antibody and incubated at 4 °C for 6 h. An appropriate amount of BSA-blocked Protein A/G magnetic beads (Thermo Scientific, 26162) was added to the mixture overnight. After the magnetic beads were cleaned twice with low-salt Tris buffer and high-salt Tris buffer, the magnetic beads were resuspended in lysate buffer, and an appropriate volume was taken for western blotting to verify the IP effect. Other magnetic beads were collected and eluted with protein K buffer at 55 °C for 30 min. Precipitated RNA was collected with an RNeasy MinElute ^®^ Cleanup Kit (Qiagen, 74204). The extracted RNA was then sent to Genevan Biotech (Shanghai, China), for sequencing.

### Data collection

Prostate cancer datasets from the TCGA database, including the gene expression profiles, somatic mutation data and copy number data, were downloaded from GDC PanCanAtlas Publications. GSE147885 (43), containing the m6A-seq dataset of a human prostate cell line, was obtained from the GEO database.

### Differential expression analysis

Differentially expressed genes between the two groups were defined with the limma (3.48.3) package (44). After log2 transformation, the standardized counts were used for differential expression analysis. Genes that met the criterion of adjusted P < 0.01 were regarded as differentially expressed between the two groups.

### Enrichment analyses

GO and KEGG enrichment analyses of DEGs were performed by clusterProfiler (4.0.5) (45). The minimum number of genes annotated by term for clustering was set to 10, and the maximum number of genes annotated for clustering was set to 500. All other parameters were set to their default settings. P values were adjusted by the Benjamini–Hochberg (BH) method. The results were considered statistically significant at a false discovery rate (FDR) <0.05.

### Somatic copy number alteration analysis

Copy number files (broad.mit.edu_pancan_genome_wide_snp_6_whitelisted. SEG) were downloaded from GDC PanCanAtlas publications for analysis. GISTIC 2.0 software was used to analyze CN files with the command line parameters indicated in the GDC documentation (46).

### m6A data analysis

GSE147885 MeRIP-seq data were downloaded, and the data type was converted from SRA to fastq. The reads were trimmed for quality control. Bowtie2 (47) was used to compare reads with rRNA, and the unmapped reads were retained for further analysis. Hisat2 (48) was used to annotate the remaining reads according to the hg38 human genome assembly (GENCODE, GRCh38.p13). Peak calling was carried out using exomePeak2.

### DNA mutations and copy number alterations of m6A regulators and their correlation with their expression

In the cBioPortal database (https://www.cbioportal.org/), three datasets, including prostate adenocarcinoma (TCGA, Cell 2015), prostate adenocarcinoma (TCGA, Firehose Legacy) and prostate adenocarcinoma (TCGA, PanCancer Atlas), were applied to detect DNA mutations and copy number alterations of all m6A regulators. All cases in the above three datasets were included for DNA mutation and copy number alteration analysis. Then, the correlation analysis for every m6A regulator was performed between mRNA expression [e.g., mRNA expression (RNA Seq V2 RSEM) (log2(value + 1)] and copy number (e.g., capped relative linear copy-number values). The expression {e.g., mRNA expression (RNA Seq V2 RSEM) [log2(value + 1)]} of every m6A regulator was compared between mutation and no mutation.

### Expression and mutation analysis for pan-cancers

Modules including “Gene_DE”, “Gene_mutation” and “Gene_Corr” in the function “cancer exploration” in the Timer 2.0 database (http://timer.cistrome.org/) were applied to detect ELAVL1 expression, its relationship with its mutation and the correlation between ELAVL1 and other m6A regulators in pan-cancers.

### Statistical analysis

Statistical analyses were performed using R software (version 4.1.1) or SPSS 22.0 (SPSS Inc., Chicago, IL, United States). Student’s t test was used to analyze expression differences between mutation and no mutation by SPSS 22.0. Pearson’s correlation analysis (correlation coefficient: 0.8-1.0, very strong correlation; 0.6-0.8, strong correlation; 0.4-0.6, medium correlation; 0.2-0.4, weak correlation; 0.0-0.2, very weak correlation.) was performed to determine the correlation between the two variables by R software. Progression-free interval (PFI) analysis *via* the Kaplan–Meier method was performed using Log Rank by R software. Statistical significance was set at p < 0.05.

## Results

### Multiomics characterization of m6A regulators in PCa

**Figure 1:**
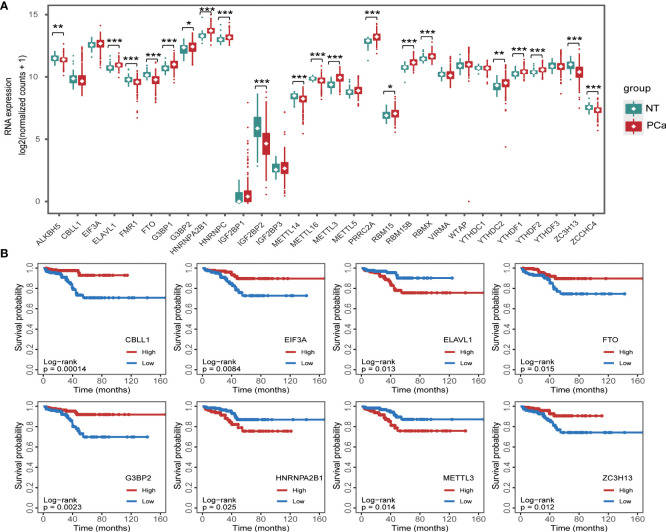
RNA m6A regulator expression in prostate cancer **(A)** and its relationship with progression-free interval **(B)**. *p < 0.05, **p < 0.01, ***p < 0.001.

Accumulating studies have shown that dysregulation of m6A regulators is associated with tumorigenesis (17–21). To clarify the expression patterns of m6A regulators in PCa, we analyzed the expression of m6A regulators in the PCa dataset from the TCGA database (**Figure 1A**) and found that the expression levels of 21 m6A regulators were significantly dysregulated. Subsequently, we analyzed the effects of these m6A regulators on the survival of PCa patients (**Figure 1B**) and showed that 8 m6A regulators, CBLL1, EIF3A, ELAVL1, FTO, G2BP2, HNRNPA2B1, METTL3, and ZC3H13, were related to the PFI of patients.

**Figure 2:**
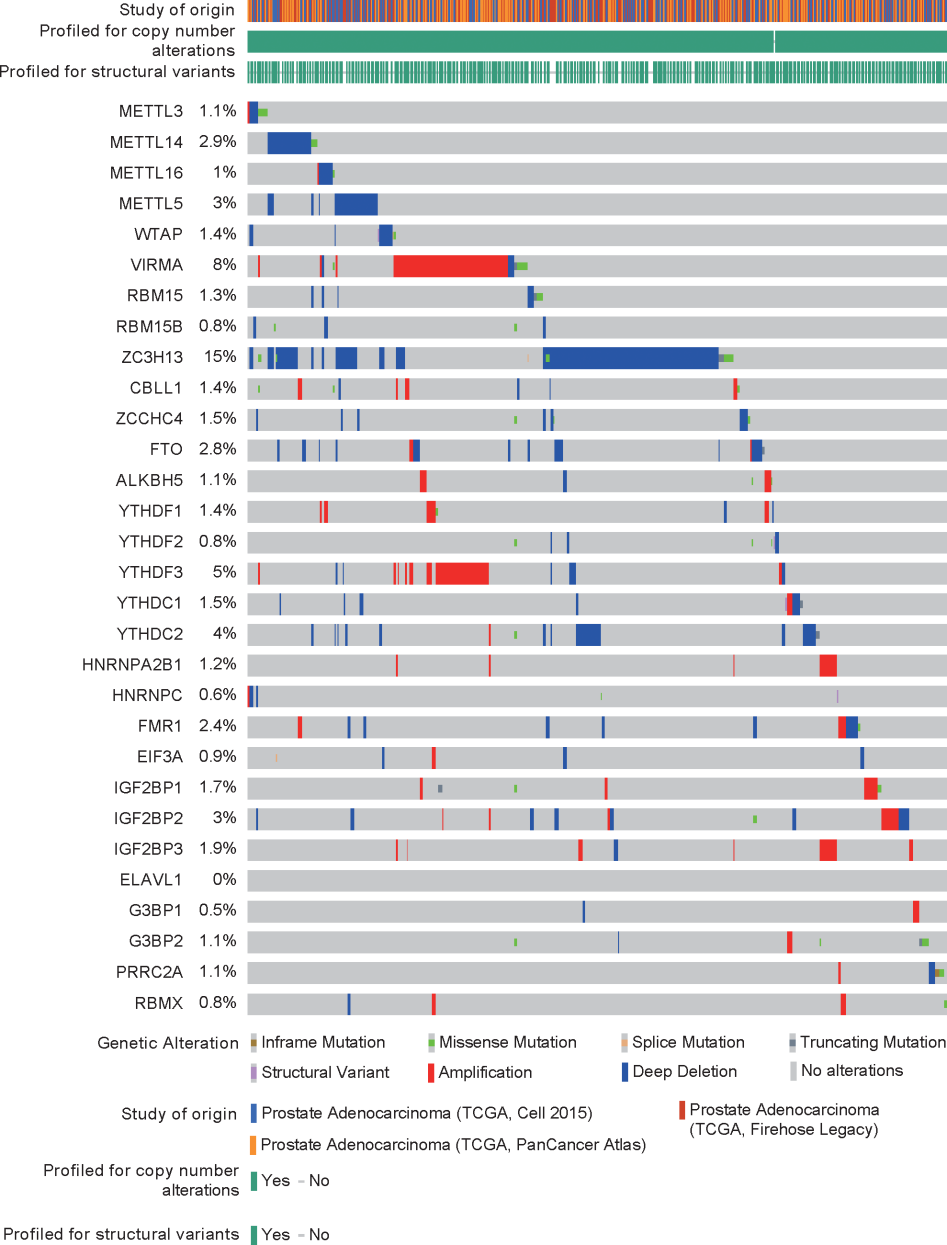
Gene mutations and copy number variants of RNA m6A regulators in prostate cancer.

DNA mutations or copy number alterations can affect gene transcription. We analyzed the mutation status of each m6A regulator in PCa in the cBioPortal database (**Figure 2**). The results showed that except for ELAVL1, the m6A regulators have mutations or copy number variations (CNVs) in PCa, and CNVs are predominant. Among these CNVs of m6A regulators, CNVs of ZC3H13 were largest. Then, we analyzed the correlation between DNA methylation (or copy number) and m6A regulator expression. The expression levels of most m6A regulators were related to their DNA methylation and copy number (**Figures S1**–**2**).

### ELAVL1 is highly expressed in PCa and is associated with tumor proliferation

**Figure 3:**
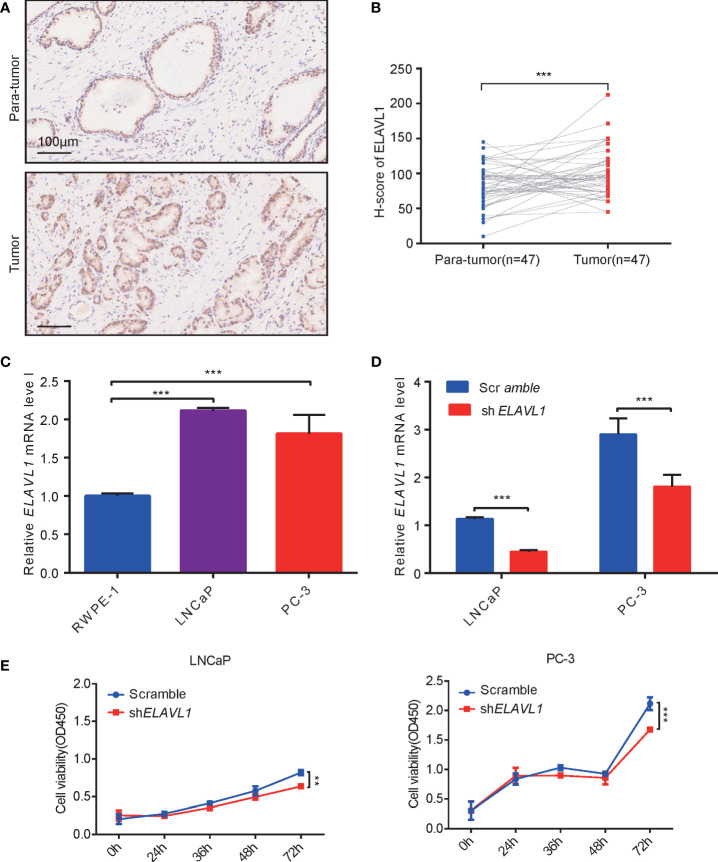
ELAVL1 promoted prostate cancer growth. **(A)** Immunohistochemistry for ELAVL1 expression in samples of prostate cancer; **(B)** H-score of ELAVL1 for immunohistochemistry of prostate cancer samples; **(C)** ELAVL1 expression in cell lines of prostate cancer; **(D)** RT–qPCR for ELAVL1 knockdown in prostate cancer cells; **(E)** CCK-8 for detecting prostate cancer proliferation after ELAVL1 knockdown. **p < 0.01, ***p < 0.001.

ELAVL1 has been reported to be involved in tumor occurrence (27–29). In 47 PCa samples, we found that ELAVL1 was indeed more highly expressed in PCa than in para-tumor tissues (**Figures 3A**, **B**). This was consistent with previous TCGA analysis data. Subsequently, we observed higher levels of ELAVL1 mRNA in LNCaP and PC-3 cells than in RWPE-1 cells (**Figure 3C**). PCa cell proliferation was inhibited after ELAVL1 knockdown (**Figures 3D**, **E**).

### Characterization of PCa with high and low ELAVL1 expression

**Figure 4:**
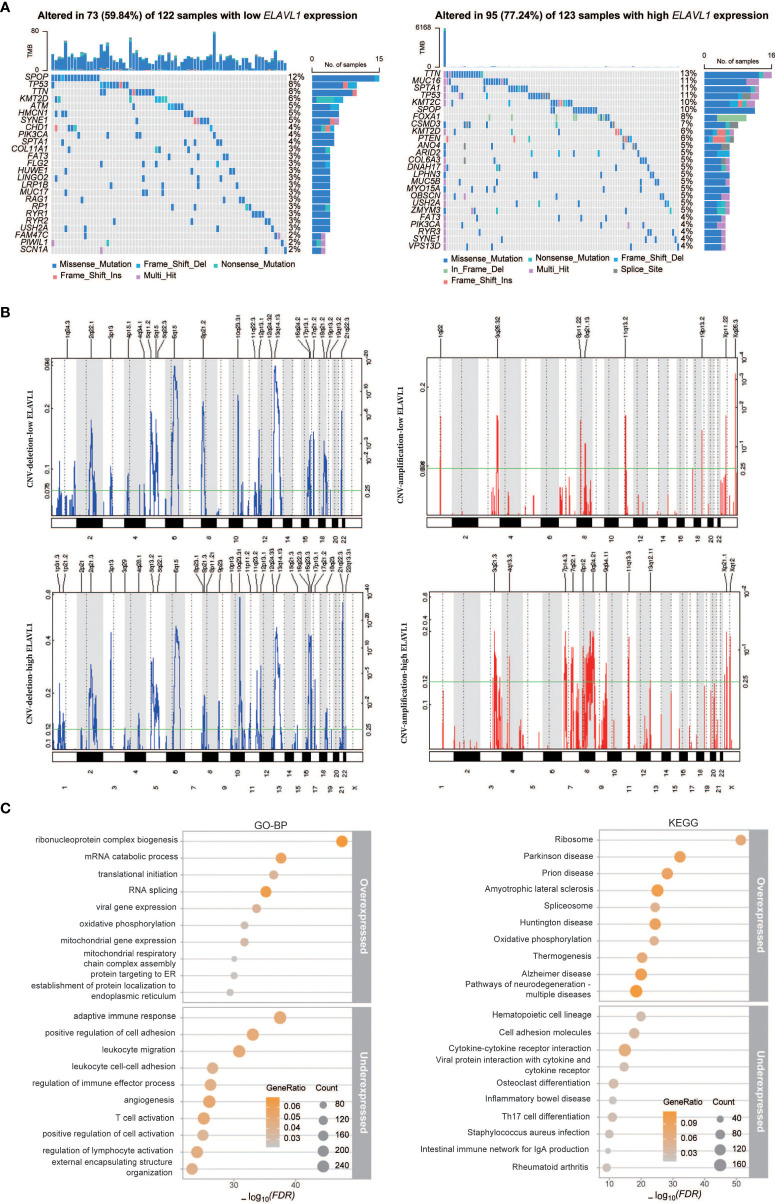
Differences in the characteristics of PCa patients with low and high ELAVL1 expression. **(A)** Landscape of gene mutations in the low-ELAVL1 and high-ELAVL1 groups; **(B)** GISTIC q-values for deletions and amplifications plotted across the genome; **(C)** GO-BP and KEGG enrichment analysis of differentially expressed genes between the low and high groups.

To evaluate the differences between high and low ELAVL1 expression levels in PCa, we divided the PCa cases in the TCGA database into PCa with low ELAVL1 expression and PCa with high ELAVL1 expression. We found that the two groups differed significantly in terms of DNA mutation profile (**Figure 4A**) and CNVs (**Figure 4B**). The number of gene mutations in the high-ELAVL1 group was significantly higher than that in the low-ELAVL1 group. The types of genetic mutations were not the same in the two groups. In addition to some common mutated genes, there were unique mutated genes in each group, such as MUC16 and KMT2C in the high-ELAVL1 group and ATM and HMCN1 in the low-ELAVL1 group. In addition, some common mutated genes also had significantly different mutation frequencies between the two groups. For example, SPTA1 had a high mutation frequency in the high-ELAVL1 group and a low mutation frequency in the low-ELAVL1 group. Then, we analyzed the somatic copy number alterations (SCNAs) in the low-ELAVL1 and high-ELAVL1 groups and found that the regions of significant focal SCNAs, including amplification and deletion, were almost completely different between the two groups. The numbers of regions with expansions and deletions were greater in the high-ELAVL1 group than in the low-ELAVL1 group.

To further analyze the expression signatures of the two groups, we performed differential expression analysis, followed by BP analysis and KEGG analysis of the identified DEGs (**Figure 4C**). The BP analysis showed that the upregulated genes were mainly enriched in terms related to RNA metabolism, such as translation and splicing, and the downregulated genes were mainly enriched in immune-related terms. KEGG analysis showed that upregulated genes were also enriched in ribosome and splicing signaling pathways, while downregulated genes were enriched in immune-related signaling pathways.

### ELAVL1 is involved in the regulation of m6A regulators in PCa

**Figure 5:**
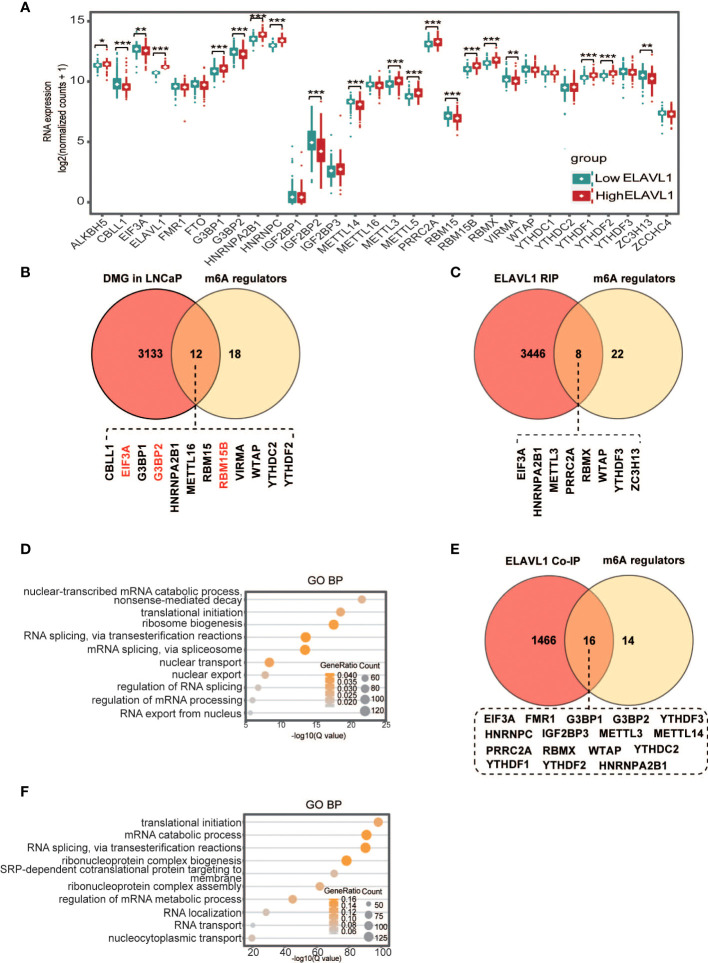
Interactions between ELAVL1 and other m6A regulators. **(A)** Differentiated expression of m6A regulators between the low- and high-ELAVL1 groups; **(B)** Venn diagram for differentially expressed m6A genes in LNCaP cells and m6A regulators; **(C)** Venn diagram for ELAVL1 RIP and m6A regulators; **(D)** GO analysis for ELAVL1 RIP; **(E)** Venn diagram for ELAVL1 co-IP and m6A regulators; **(F)** GO analysis for ELAVL1 co-IP. *p < 0.05, **p < 0.01, ***p < 0.001.

As noted above, the highly expressed genes in the high-ELAVL1 group were enriched in RNA metabolism. It is known that m6A regulates RNA metabolism, and dysregulated m6A affects RNA metabolism to promote tumor development (17–21). Thus, we asked whether there is a relationship between ELAVL1 and other m6A regulators, including ALKBH5, CBLL1, EIF3A, FMR1, FTO, G3BP1, G3BP2, HNRNPA2B1, HNRNPC, IGF2BP1, IGF2BP2, IGF2BP3, METTL14, METTL16, METTL3, PRRC2A, RBM15, RBM15B, RBMX, VIRMA, WTAP, YTHDC1, YTHDC2, YTHDF1, YTHDF2, YTHDF3, ZC3H13 and ZCCHC4. The expression levels of 20 m6A regulators were significantly different between the high- and low-ELAVL1 groups (**Figure 5A**), and correlation analysis showed that there was a significant correlation between the expression of ELAVL1 and that of other m6A regulators (**Figure S3**). In addition, different m6A regulators appeared in SCNAs, including both amplifications and deletions, associated with the high- and low-ELAVL1 groups.

Several studies have found that ELAVL1 is involved in regulating tumorigenesis as an m6A binding protein (33, 35). To clarify how ELAVL1 affects other m6A regulators, we hypothesized that some m6A regulators might have m6A sites. After reanalysis of the GSE147885 dataset in the GEO database (**Supplementary File 1**), we found that the m6A levels of 12 m6A regulators were significantly altered in LNCaP cells compared with RWPE-1 cells, with increased m6A levels for RBM15B, G3BP2, and EIF3A and decreased m6A levels for the other m6A regulators (**Figure 5B**). This result indicated that some m6A regulators indeed have m6A sites. To determine whether ELAVL1 binds to these m6A regulators *via* m6A, we performed ELAVL1 RIP-seq and found that ELAVL1 indeed interacted with 8 m6A regulators, namely, EIF3A, METTL3, PRRC2A, RBMX, HNRNPA2B1, WTAP, YTHDF3 and ZC3H13 (**Figure 5C**; **Supplementary File 2**). IgV plots showed that three m6A regulators, namely, WTAP, ZC3H13 and EIF3A, had marked m6A peaks in LNCaP cells (**Figure S4**). Through the POSTAR3 database (http://111.198.139.65), we obtained potential ELAVL1 binding sites on the above three m6A regulators, and the sites within the m6A peak were considered ELAVL1 binding sites on the three m6A regulators in LNCaP cells (**Supplementary File 3**). Additionally, mutation sites of the three regulators in **Figure 2** (**Supplementary File 4**) obtained from the cBioPortal database (https://www.cbioportal.org/) did not appear in the ELAVL1 binding site on the three regulators in LNCaP cells. Finally, GO analysis showed that ELAVL1-immunoprecipitated mRNAs were associated with RNA metabolism functions, including RNA decay, translation, splicing, and nuclear export (**Figure 5D**). Previous studies have shown that PCa development is associated with RNA metabolism due to m6A imbalance (49–51). Therefore, we speculate that ELAVL1 in PCa may be involved in tumor development by regulating the RNA metabolism of m6A regulators.

Studies have found that ELAVL1 interacts with YTHDC1 and IGF2BP1 to play a synergistic regulatory role in tumors (36, 37). We asked whether ELAVL1 also interacts with other regulators. We performed ELAVL1 coimmunoprecipitation combined with mass spectrometry analysis in PCa cells, and the results showed that ELAVL1 interacts with 16 m6A regulators (**Figure 5E**, **Supplementary File 5**). In addition, GO analysis of molecules immunoprecipitated by ELAVL1 showed that these molecules were indeed enriched in gene sets related to RNA metabolism, including translation and alternative splicing (**Figure 5F**).

### ELAVL1 in other tumors

**Figure 6:**
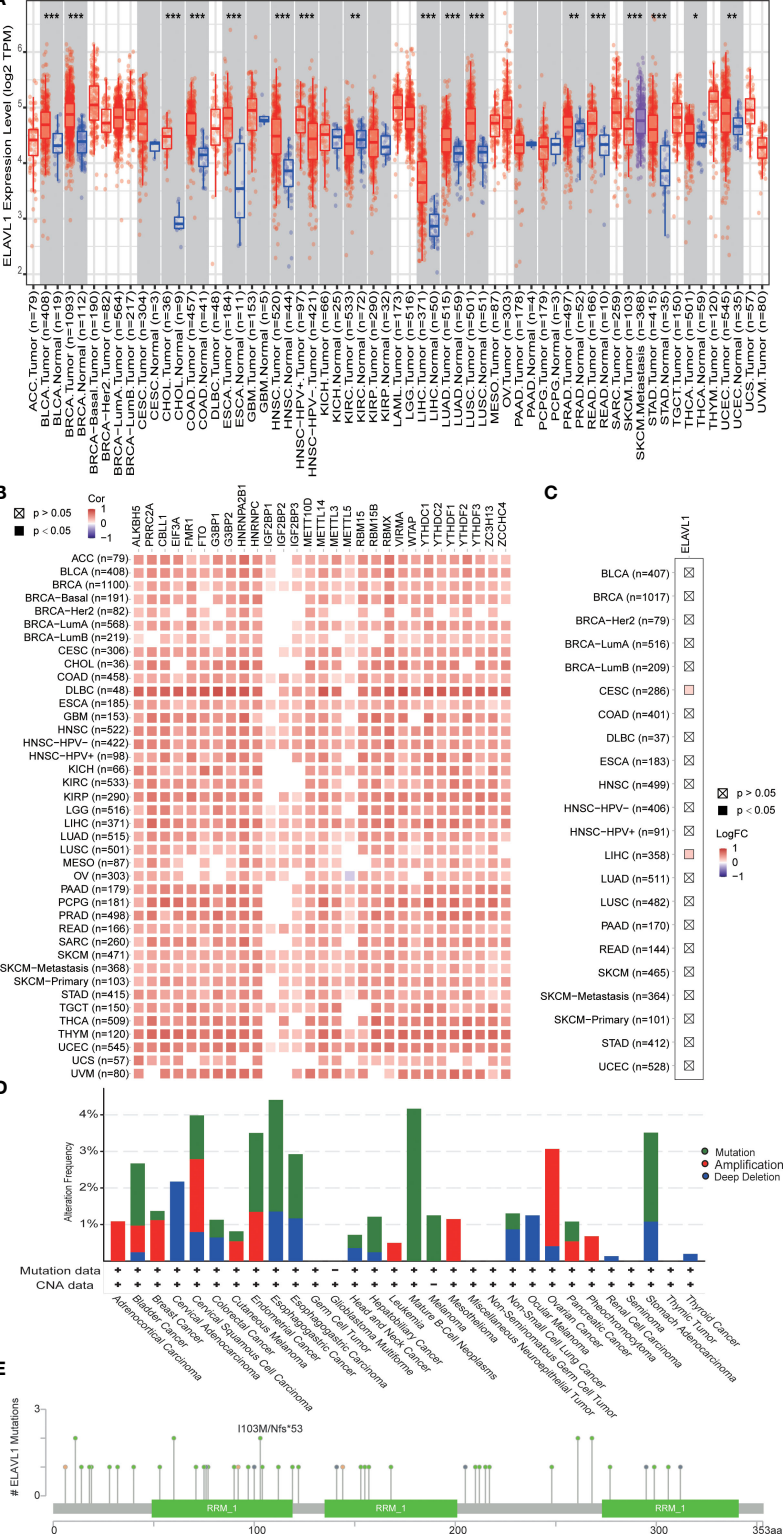
ELAVL1 in various types of tumors. **(A)** ELAVL1 expression in tumors; **(B)** correlation between ELAVL1 and other m6A regulators in tumors; **(C)** ELAVL1 mutation in tumors in the TIMER 2.0 database; **(D)** ELAVL1 mutation and copy number variants in tumors in the CBioPortal database; **(E)** landscape of ELAVL1 mutation sites. *p < 0.05, **p < 0.01, ***p < 0.001.

In our study, ELAVL1 was found to be highly expressed in prostate cancer. Knockdown of ELAVL1 can inhibit the proliferation of prostate cancer. In addition, ELAVL1 interacted with other m6A regulators. We wanted to further understand the characteristics of ELAVL1 in other tumors. In TCGA data, ELAVL1 was found to be highly expressed in almost all tumors, indicating that high ELAVL1 expression is closely related to tumor development (**Figure 6A**). In addition, in almost all tumors, there was a significant correlation between the expression of ELAVL1 and other m6A regulators (**Figure 6B**), indicating that the interaction between ELAVL1 and other m6A regulators is a common phenomenon in tumors. In addition, in the TIMER2.0 database, we found no significant difference in the mutation frequency of ELAVL1 in almost all tumors (**Figure 6C**). In the cBioPortal database, we found a low frequency of ELAVL1 mutation and CNV in almost all tumors (**Figure 6D**). The low-frequency ELAVL1 mutation sites are shown in **Figure 6E**.

## Discussion

PCa is a common tumor in elderly men. Although there are various clinical treatments, the mortality rate is still high. Moreover, the detailed pathogenesis of PCa is still unclear. The findings related to m6A have opened up new directions for the study of the pathological mechanisms of the disease. m6A dysregulation has been confirmed to be related to tumor occurrence and drug resistance (17–21, 52, 53), and many m6A regulators are significantly dysregulated in various tumors (17–21). Corresponding bioinformatics analysis studies have also found DNA mutations and CNVs of m6A regulators (54). A previous bioinformatic study of m6A regulators in PCa showed that the expression of most m6A regulators was dysregulated and that some regulators were related to survival (55). Similarly, we also found that multiple m6A regulators were dysregulated in PCa. However, m6A regulators related to the progression-free interval, including CBLL1, EIF3A, ELAVL1, FTO, G2BP2, HNRNPA2B1, METTL3, and ZC3H13, were partially different from those including RBM15B, METTL3, HNRNPA2B1, RBMX, YTHDF1 and HNRNPC in a previous bioinformatic study (55). This discrepancy was due to the different samples included in our study. Additionally, in our study, most of the m6A regulators exhibited gene mutations and CNVs, consistent with previously published findings (56). The dysregulated expression of multiple m6A regulators in PCa was related to DNA methylation levels and copy number. DNA mutations, changes in methylation and CNVs are important etiologies of tumor development. We believe that alterations in m6A levels in PCa are associated with alterations of these m6A regulators at the DNA level. The results of this study are similar to those related to m6A in other tumors (57).

Before ELAVL1 was recognized as an m6A regulator, ELAVL1, as a traditional RNA-binding protein, was found to be involved in the occurrence and progression of almost all tumors (58, 59). With the development of m6A research, ELAVL1, as an m6A regulator, was found to interact with other m6A regulators to regulate RNA metabolism (36, 37). Therefore, among many m6A regulators, we focused on the *ELAVL1* gene. In addition, cumulative studies have found that ELAVL1 is not only related to tumor occurrence and progression but also to radiotherapy resistance and chemotherapy resistance (27–29, 58). These findings suggest that ELAVL1 is an important target for tumor treatment and that disrupting ELAVL1 expression in tumors is expected to inhibit tumors or improve the therapeutic response. In PCa, it was reported that normal prostate epithelium had mild to moderate predominantly nuclear immunostaining of ELAVL1 and both higher cytoplasmic and nuclear expression in cancer cells (38). Similarly, our study also showed that compared with para-tumor tissue, PCa tumor tissue had stronger ELAVL1 staining and a higher H-score. High ELAVL1 was found to be related to tumor proliferation. We further analyzed the differences in transcripts between high- and low-ELAVL1 PCa and found that highly expressed genes in high-ELAVL1 PCa were enriched in RNA metabolism. At the same time, we found significant differences in the expression of m6A regulators between the two groups, and correlation analysis also showed that ELAVL1 expression was significantly correlated with the expression of other m6A regulators. The above results led us to speculate that ELAVL1 promotes PCa growth and may be related to the regulation of other m6A regulators. For example, METTL3 has been found to promote PCa development, and our study shows that METTL3 expression is related to ELAVL1 expression. Therefore, ELAVL1 may regulate PCa development by affecting METTL3.

In studies on m6A and tumors, ELAVL1, as an m6A reader, was found to regulate RNA stability in an m6A-dependent fashion (33, 35). In this study, we found that compared with those in RWPE-1 cells, the m6A levels on some m6A regulators were either upregulated or downregulated in PCa cells. These m6A regulators with altered m6A levels are considered to be m6A modifications. We also further found that m6A regulators can bind to ELAVL1, so we believe that ELAVL1 may regulate the RNA metabolism of some m6A regulators through m6A modification in PCa. Previous studies have found that ELAVL1 can interact with proteins such as YTHDC1 and IGF2BP1 and can synergistically promote RNA stabilization (36, 37). We also found that ELAVL1 can interact with 16 m6A regulators in PCa. The above findings suggest that there is a close relationship between ELAVL1 and other m6A regulators in PCa; further experimental studies are needed to uncover the detailed role of ELAVL1.

Several studies have found a tumor-promoting effect of ELAVL1 in various tumors (27–29). Our study also found that ELAVL1 expression was upregulated in almost all tumors. However, the mutation frequency of *ELAVL1* in tumors was low, indicating that the upregulated expression of ELAVL1 may not be a cause of tumorigenesis. In addition, our study also found an interaction between ELAVL1 and m6A regulators in almost all tumors. Cumulative studies have confirmed that m6A imbalance is closely related to tumors (13–16). Therefore, we believe that although high expression of ELAVL1 is not the cause of tumorigenesis, it may be one of the key molecules in this process.

In conclusion, our study shows that the expression of m6A regulators is significantly deregulated and that these m6A regulators exhibit DNA mutations and CNVs in PCa. In addition, ELAVL1 is highly expressed in PCa and can promote tumor proliferation. ELAVL1 may rely on m6A to participate in the regulation of RNA metabolism of m6A regulators or interact with m6A regulators to play a tumor-promoting role.

## Data availability statement

The original contributions presented in the study are included in the article/**Supplementary Material**. The raw RIP-seq data presented in this study have been deposited in the Genome Sequence Archive in BIG Data Center, Beijing Institute of Genomics (BIG), Chinese Academy of Sciences, under accession number CRA007447 that is accessible at https://bigd.big.ac.cn. Further inquiries can be directed to the first author and corresponding authors.

## Ethics Statement

The studies involving human prostate cancer samples were reviewed and approved by the Ethics Committee of Shanghai Outdo Biotech Company. Written informed consent was obtained from all participants for their participation in this study.

## Author contributions

ZW, LHJ and DW had full access to all of the data in the study and take responsibility for the integrity of the data, the accuracy of the data analysis, and the critical revision of the manuscript for important intellectual content. ZLC, HX, GB and HJH took responsibility for the concept, design, data analysis, and paper written. All authors contributed to the article and approved the submitted version.

## Funding

This study was funded by the grants from National Natural Science Foundation of China (Grant No. 82170788) and Project of biobank from Shanghai Ninth People’s Hospital, Shanghai Jiao Tong University School of Medicine (Grant No. YBKB201904).

## Acknowledgments

We thank Genefund Biotech (Shanghai, China) for assistance in the experiments and data analysis.

## Conflict of Interest

The authors declare that the research was conducted in the absence of any commercial or financial relationships that could be construed as a potential conflict of interest.

## Publisher’s note

All claims expressed in this article are solely those of the authors and do not necessarily represent those of their affiliated organizations, or those of the publisher, the editors and the reviewers. Any product that may be evaluated in this article, or claim that may be made by its manufacturer, is not guaranteed or endorsed by the publisher.

## Supplementary material

Supplementary Figure 1 Correlation between the expression level and copy number of m6A regulators.

Supplementary Figure 2 Comparison of m6A regulator expression between mutation and no mutation. *p<0.05, ***p<0.001.

Supplementary Figure 3 Correlation between ELAVL1 expression and the expression of other m6A regulators.

Supplementary Figure 4 m6A peaks of m6A regulators and ELAVL1-binding sites in LNCaP cells.

Supplementary file 1 Differential methylation analysis between LNCaP and RWPE-1 cells from GSE147885.

Supplementary file 2 ELAVL1-immunoprecipitated RNAs in LNCaP cells.

Supplementary file 3 ELAVL1 binding sites in the m6A peak of m6A regulators in LNCaP cells.

Supplementary file 4 List of mutation sites of WTAP, ZC3H13 and EIF3A in obtained from the cBioPortal database.

Supplementary file 5 ELAVL1-immunoprecipitated proteins by mass spectrometry in LNCaP cells.
