# Supplementary figures and images for: ELAVL1 promotes prostate cancer progression by interacting with other m6A regulators
Source: Front Oncol. 2022 Aug 1;12:939784. doi: 10.3389/fonc.2022.939784 (PMC9376624; doi:10.3389/fonc.2022.939784)

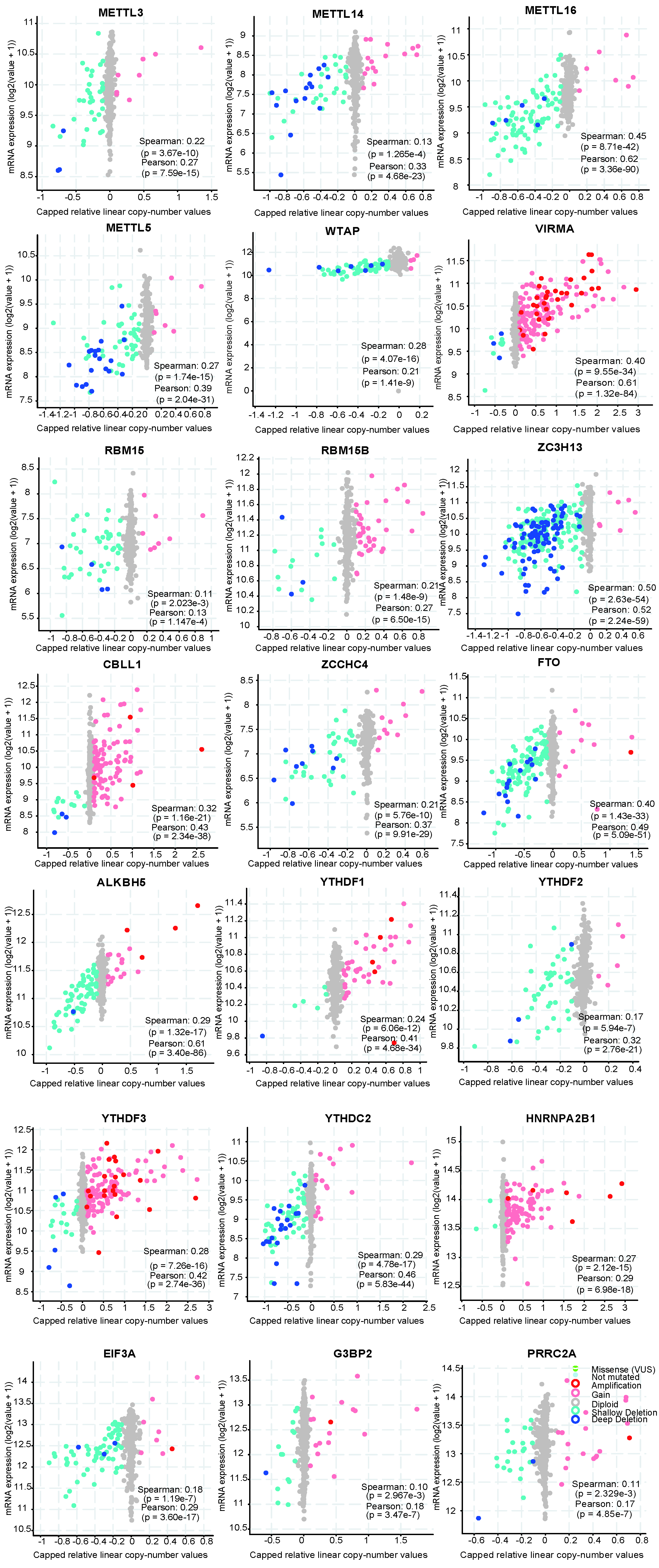

Supplement: Supplementary Figure 1 — Correlation between the expression level and copy number of m6A regulators. [file Image_1.tiff]

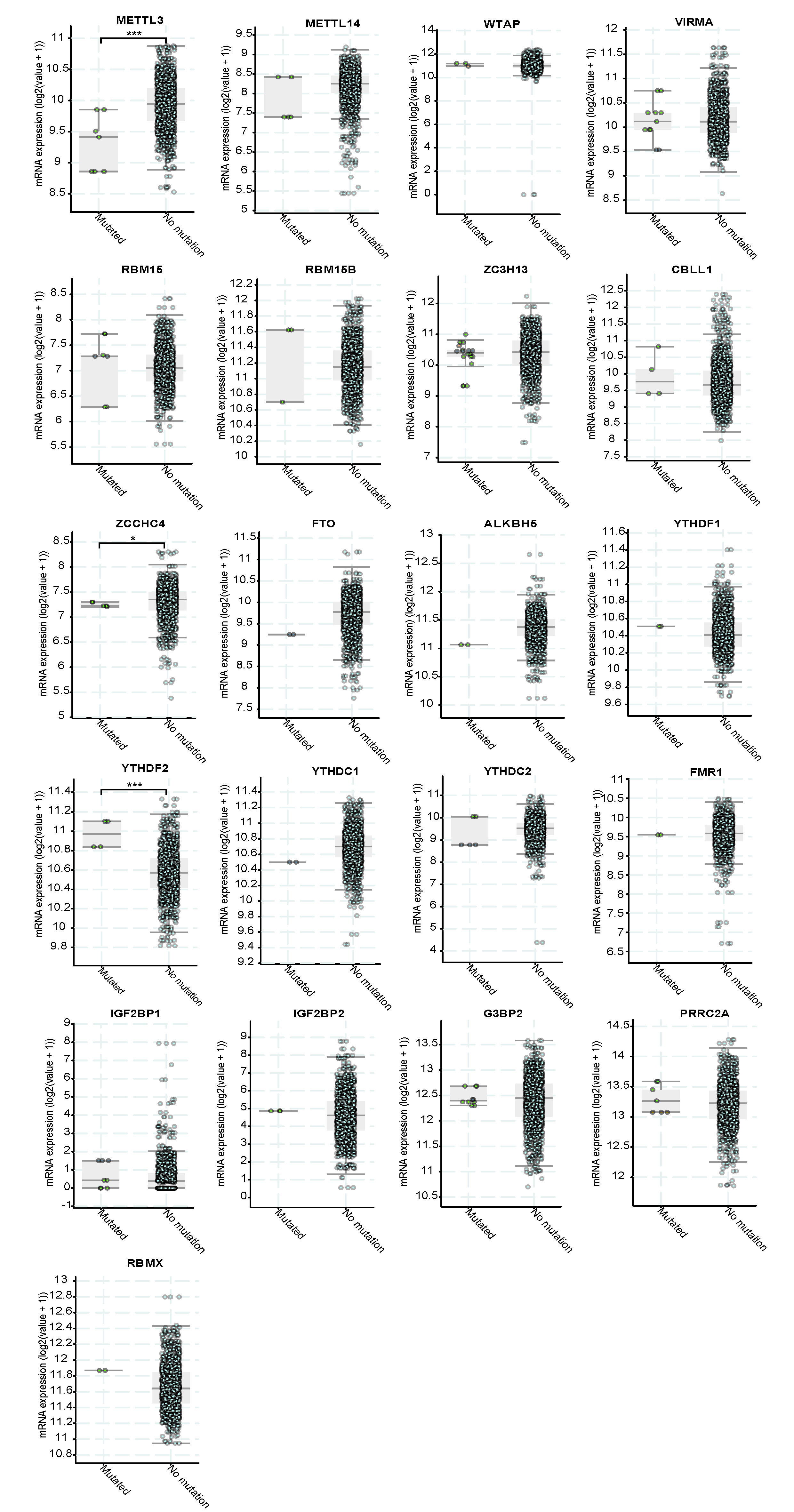

Supplement: Supplementary Figure 2 — Comparison of m6A regulator expression between mutation and no mutation. *p<0.05, ***p<0.001. [file Image_2.tiff]

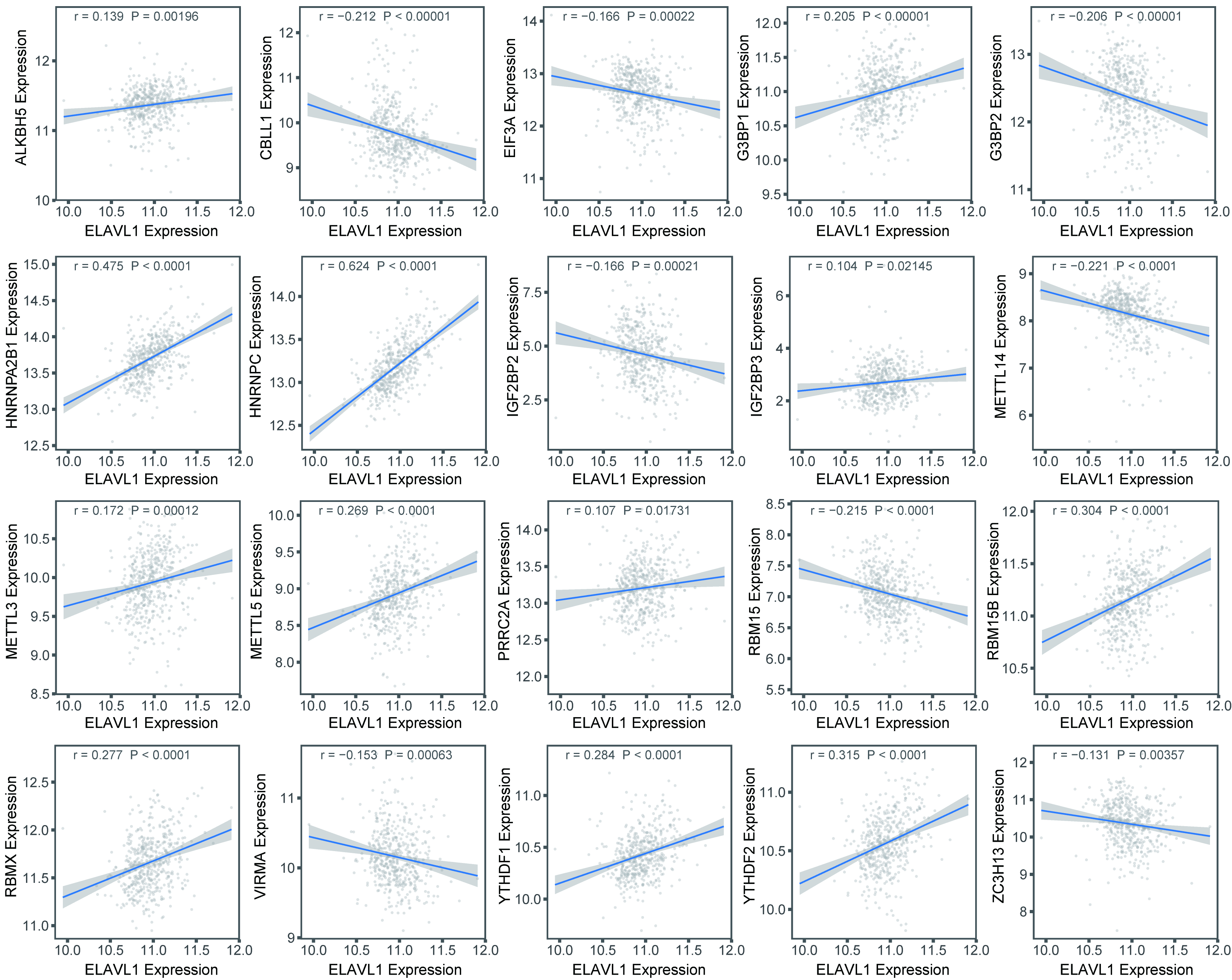

Supplement: Supplementary Figure 3 — Correlation between ELAVL1 expression and the expression of other m6A regulators. [file Image_3.tif]

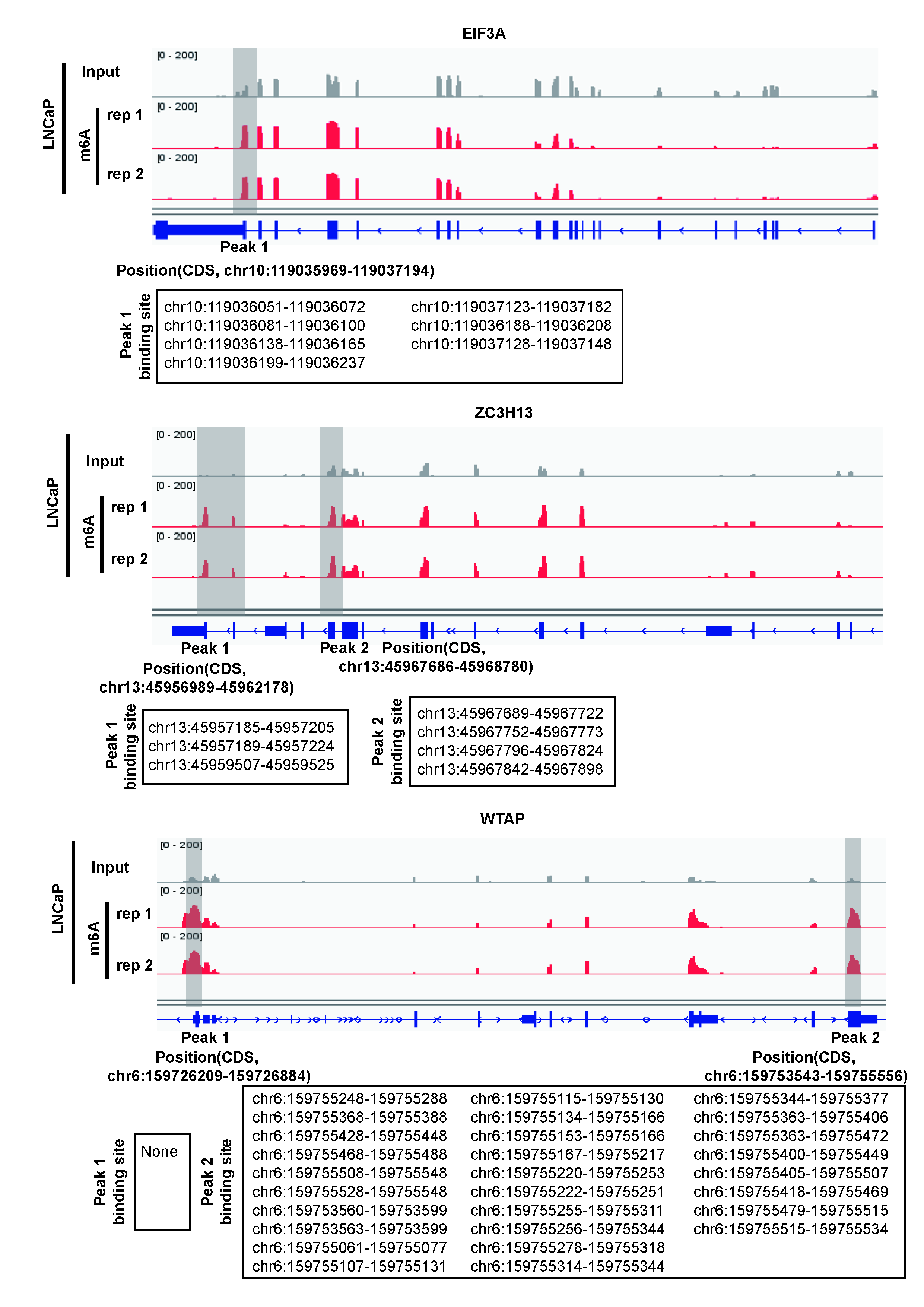

Supplement: Supplementary Figure 4 — m6A peaks of m6A regulators and ELAVL1-binding sites in LNCaP cells. [file Image_4.tiff]
